# Prevention of UVB-Induced Photoaging by an Ethyl Acetate Fraction from *Allomyrina dichotoma* Larvae and Its Potential Mechanisms in Human Dermal Fibroblasts

**DOI:** 10.3390/ijms25147850

**Published:** 2024-07-18

**Authors:** Kyong Kim, Chae-Eun Kim, Dong-Jae Baek, Eun-Young Park, Yoon Sin Oh

**Affiliations:** 1 Department of Food and Nutrition, Eulji University, Seongnam 13135, Republic of Korea; kim_kyong@hanmail.net (K.K.); kce0223@naver.com (C.-E.K.); 2 College of Pharmacy, Mokpo National University, Mokpo 58554, Republic of Korea; dbaek@mokpo.ac.kr (D.-J.B.); parkey@mokpo.ac.kr (E.-Y.P.)

**Keywords:** skin, aging, *Allomyrina dichotoma* larvae, ethyl acetate fraction, antioxidant

## Abstract

*Allomyrina dichotoma* larvae (ADL) is an insect type that is used ethnopharmacologically to treat various diseases; however, its use as an antiaging treatment has not been widely studied. Previously, we found that an ethyl acetate (EA) fraction derived from an ADL extract (ADLE) has a high polyphenol content and antioxidant properties. In this study, we identified the underlying molecular mechanism for the protective effect of the EA fraction against UVB-induced photodamage in vitro and ex vivo. UVB treatment increased intracellular reactive oxygen species levels and DNA damage; the latter of which was significantly decreased following cotreatment with the EA fraction. Biological markers of aging, such as p16^INK4a^, p21^WAF1^, and senescence-associated β-gal levels, were induced by UVB treatment but significantly suppressed following EA-fraction treatment. UVB-induced upregulation of matrix metalloproteinase (MMP)-1 and downregulation of COL1A1 were also reversed by EA-fraction treatment in both cells and a 3D skin model, which resulted in increased keratin and collagen deposition. Moreover, EA-fraction treatment inhibited the phosphorylation of MAPKs (p38, ERK, and JNK) and nuclear factor (NF-)-kB and decreased the levels of inflammatory cytokines in UVB-treated cells. The results indicate that an EA fraction from ADLE ameliorates UVB-induced degradation of COL1A1 by inhibiting MMP expression and inactivating the MAPK/NF-κB p65/AP-1 signaling pathway involved in this process.

## 1. Introduction

In general, the condition of the external skin, which protects the whole body, reflects the degree of health and aging. Skin aging is characterized by wrinkles, sagging, roughness, and dryness, which naturally appear with age but are also promoted by external factors, with ultraviolet (UV) radiation being the most important factor [1]. A certain amount of UV exposure is indispensable for humans in order to synthesize vitamin D in the skin. However, overexposure to UVB (ultraviolet B radiation), which has various biological effects at wavelengths corresponding to ultraviolet rays of 280–320 nm, damages DNA, directly and through the generation of reactive oxygen species (ROS) in skin fibroblasts. This disrupts the antioxidant defense system, induces oxidative stress, and increases the activity of matrix metalloproteinase (MMP), resulting in photoaging [2,3].

Research on the mechanism of skin photoaging has demonstrated that ascorbic acid, an antioxidant, donates two electrons and scavenges ROS [4]. It protects the skin by promoting collagen synthesis; suppressing MMP-1, a collagen-degrading enzyme; and providing photoprotection against ultraviolet rays and inflammation [5]. Organically synthesized vitamin derivatives are primarily available commercially; however, they have an unstable molecular structure. Therefore, when applied to the skin in large amounts, they can cause skin irritation and hypersensitivity-based reactions, depending on a person’s constitution [6]. Therefore, the identification of natural functional materials with fewer side effects will be useful to prevent skin aging [7,8,9].

Insects have evolved unique physiological control mechanisms and defense systems to survive inhospitable natural environments. They account for approximately 80% of all animalia on Earth, excluding the sea [10,11]. Recently, insects have been proposed as a natural antioxidant treatment used to remove active oxygen as well as a potential food source [12,13]. One edible insect form, *Allomyrina dichotoma* larvae (ADL), has long been used as a natural pharmacological ingredient in traditional Asian medicine. Various in vitro and in vivo studies have demonstrated that it has beneficial physiological properties, including anti-hepatic fibrosis, anti-tumor, anti-inflammatory, anti-obesity, and anti-neuropathy effects [14,15,16,17].

Recently, we found that total ADL extract (ADLE) attenuated free fatty acid-induced lipotoxicity in pancreatic beta cells, and that its antioxidant activity was an important mechanism [18]. Moreover, an ethyl acetate extract (EA) was fractionated from ADLE and found to have a high polyphenol content and 2,2-diphenyl-1-picrylhydrazyl (DPPH) radical scavenging activity [19]. Therefore, a decrease in oxidative stress levels may be a key mechanism in the prevention of aging. Based on previous studies, we examined the protective effect of an EA fraction prepared from ADLE on the expression of UVB-induced aging-related proteins, inflammatory factors, and apoptosis signaling pathways, in vitro and in a 3D skin model.

## 2. Results

### 2.1. Cytoprotective Effect of the EA Fraction against UVB-Irradiated Human Dermal Fibroblast (HDF) Cells

**Figure 1:**
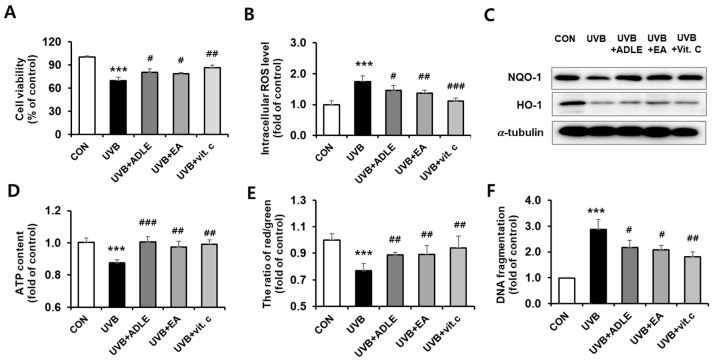
Cytoprotective effects of the ethyl acetate fraction on UVB-irradiated HDF cells. The cells were treated for 24 h with 100 µg/mL of ADLE, 100 µg/mL of EA, and 10 µM of vitamin C, after 100 mJ/cm^2^ UVB irradiation. Cell viability was measured using the MTT (3-(4,5-dimethylthiazol-2-yl)-2,5-diphenyltetrazolium bromide) assay (**A**). Intracellular ROS levels were assayed by detecting the fluorescence intensity of the oxidant-sensitive fluorescent probe DCFH-DA at 485/535 nm (**B**). HO-1 and NQO1 protein expression levels were measured using Western blotting. The band intensity was measured using C.S. analyzer 4 version 2.4.5 software, and the relative quantity was calculated over α-tubulin (**C**). Luciferase-based luminescence assay for cytosolic ATP content (**D**), JC-1 staining for mitochondrial membrane potential (ΔΨm) (**E**), and quantification of apoptosis via fragmented DNA analysis using a Cell Death Detection ELISA kit (**F**). Data are presented as the mean ± SD (n = 3–5). *** *p* < 0.001 versus control (CON, untreated). ^#^ *p* < 0.05, ^##^ *p* < 0.01, and ^###^ *p* < 0.001 versus UVB treatment alone.

We determined whether an EA fraction of ADLE, previously identified as an antioxidant component, exerts cytoprotective effects. After 24 h of exposure, 100 mJ/cm^2^ UVB irradiation reduced HDF cell viability to 70%, compared with the nonirradiated control; however, 100 μg/mL of the EA fraction significantly reduced the cytotoxic effect (Figure 1A). Intracellular ROS levels increased following UVB treatment, with significantly reduced expression of antioxidant proteins such as NQO-1 and HO-1. These protein levels were increased following EA treatment (Figure 1B,C). We measured intracellular adenosine triphosphate (ATP) content and mitochondrial membrane potential (ΔΨm) using a luminescence ATP detection assay and JC-1 staining, respectively. They were found to be decreased by 10%–20% in UVB-treated cells, compared with the control (*p* < 0.001); however, this reduction was significantly reversed in EA-treated cells to the same level as vitamin C-treated cells (Figure 1D,E). The amount of DNA fragmentation was increased 3-fold (*p* < 0.001) following UVB treatment, whereas significant reductions of 42.1% (*p* < 0.05) by EA, 36.9% (*p* < 0.05) by ADLE, and 57.9% (*p* < 0.01) by vitamin C (Figure 1F) were observed. These results indicate that the EA fraction prevents cytotoxicity in UVB-exposed HDF cells.

### 2.2. Anti-Aging Effects of the EA Fraction on Senescence-Associated Secretory Phenotype-Based Biomarkers in UVB-Irradiated HDF Cells

**Figure 2:**
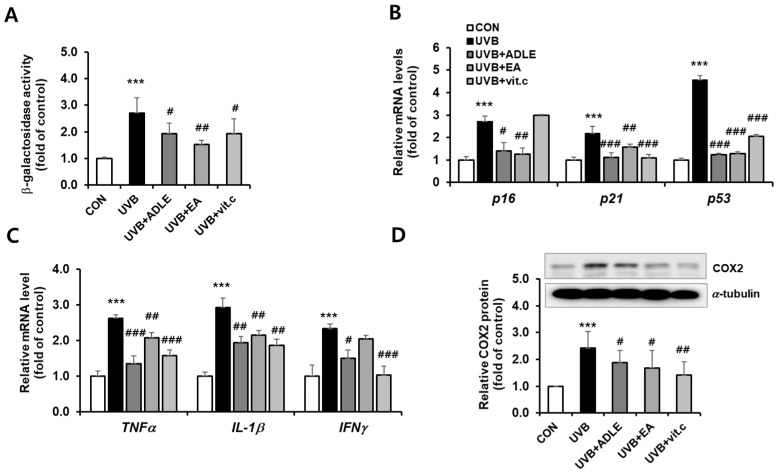
Anti-aging effects of the EA fraction on senescence-associated secretory phenotype-based biomarkers in UVB-irradiated HDF cells. Cells were treated for 24 h with 100 µg/mL of ADLE, 100 µg/mL of EA, and 10 µM of vitamin C after 100 mJ/cm^2^ UVB irradiation. Beta-galactosidase activity was assayed using a mammalian β-galactosidase assay kit (**A**). The mRNA levels of p21, p16, and p53 were determined using *q*RT-PCR. The mRNA levels were normalized with those of cyclophilin (**B**). The mRNA levels of TNFα, IL-1β, and IFNγ were determined using *q*RT-PCR. The mRNA levels were normalized with those of cyclophilin (**C**). COX2 protein expression was measured using Western blotting. The band intensity was measured using C.S. analyzer 4 version 2.4.5 software, and the relative quantity was calculated over α-tubulin (**D**). Data are presented as the mean ± SD (n = 3–5). *** *p* < 0.001 versus CON (nontreated). ^#^ *p* < 0.05, ^##^ *p* < 0.01, and ^###^ *p* < 0.001 versus UVB treatment alone.

To determine whether EA fractions exert anti-aging effects, we measured β-galactosidase activity, the expression of cell cycle-related proteins, and the production of inflammatory substances in UVB-induced HDF cells with or without ADLE and its EA fraction. Following UVB irradiation, β-galactosidase activity in HDF cells increased by 2.7-fold (*p* < 0.001) compared with untreated cells, and it was attenuated by 70.6% following EA fraction treatment (*p* < 0.01), which was higher than the inhibitory effect of ADLE, at 47.01% (Figure 2A). Next, quantitative RT-PCR was used to determine the effect of EA on the expression of cell cycle regulators, such as p21, p16, and p53. The gene expression levels of p16, p21, and p53 were significantly (*p* < 0.001) increased, by 2.7-, 2.2-, and 4.6-fold, respectively, by UVB irradiation exposure, compared with control cells (Figure 2B). The expression of these genes was significantly decreased following EA treatment. COX-2 and proinflammatory cytokines are recognized as markers of cellular senescence. Their expression was measured in UVB-irradiated HDF cells with or without EA-fraction treatment. Following UVB irradiation, the mRNA levels of TNFα, IL-1β, and interferon (IFN)γ were increased more than 2.0-fold compared to the control (CON) group (Figure 2C). This increase was reduced following EA-fraction treatment to a level similar to that of the controls (vitamin C and ADLE). The expression of COX-2 protein, an enzyme induced by cytokines, was significantly increased following UVB irradiation (*p* < 0.001), but this increase was reduced by 35.7% (*p* < 0.01) in the EA-treated group compared with the UVB-alone group (Figure 2D). These results indicate that EA suppresses UVB-induced photoaging by regulating cellular senescence mechanisms associated with the p21, p16, and p53 pathways and suppressing proinflammatory cytokine expression.

### 2.3. Regulation of COL1A1 and MMP-1 Expression via EA Treatment in UVB-Irradiated HDF Cells

**Figure 3:**
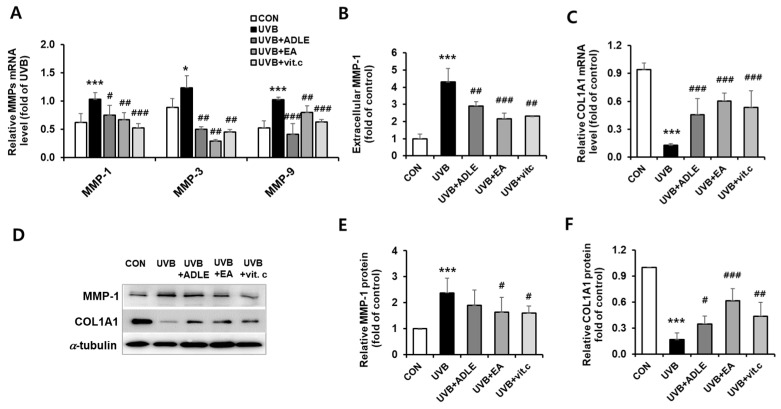
Regulation of COL1A1 and MMP-1 expression using the EA fraction in UVB-irradiated HDF cells. Cells were treated for 24 h with 100 µg/mL of ADLE, 100 µg/mL of EA, and 10 µM of vitamin C after 100 mJ/cm^2^ UVB irradiation. The mRNA expression levels of COL1A1 and MMPs were measured using *q*RT-PCR (**A**,**C**). Extracellular MMP-1 production was measured using an ELISA kit (**B**). The expression levels of COL1A1 and MMP-1 proteins were determined using Western blotting (**D**). COLIA1 and MMP-1 intensities were measured using C.S. analyzer 4 version 2.4.5 software, and relative quantities were calculated over α-tubulin (**E**,**F**). Data are presented as the mean ± SD (n = 3–5). * *p* < 0.05 and *** *p* < 0.001 versus CON. ^#^ *p* < 0.05, ^##^ *p* < 0.01, and ^###^ *p* < 0.001 versus UVB treatment alone. UVB cells were irradiated with 100 mJ/cm^2^ UVB alone; UVB + ADLE cells were treated with 100 μg/mL of ADLE after UVB irradiation; UVB + EA cells were treated with 100 μg/mL of EA fraction of ADLE after UVB irradiation; UVB + vitamin C cells were treated with 10 μM of ascorbic acid as a positive control.

Acute UV irradiation exposure induced the expression of MMPs in human skin cells (MMP-1, MMP-3, and MMP-9), and these were involved in the control of extracellular matrix (ECM) remodeling in aging [20]. As shown in Figure 3A, mRNA expression levels of collagenase MMP-1 (*p* < 0.001), MMP-3 (*p* < 0.05), and MMP-9 (*p* < 0.001) were significantly increased in UVB-irradiated HDF cells, but these expressions were ameliorated by treatment with EA fraction. As MMP-1 primarily degrades type 1 and 3 collagens, which are abundant in the skin [21], we also determined the effect of EA on UVB-induced MMP-1 secretion using enzyme-linked immunosorbent assay (ELISA). UVB irradiation of HDFs increased extracellular MMP-1 secretion by 4.3-fold compared to controls (*p* < 0.001). However, ADLE and the EA fraction significantly diminished UBV-induced MMP-1 secretion by 42.2% (*p* < 0.01) and 64.8% (*p* < 0.001), respectively, compared to the UVB-treated group (Figure 3B). Additionally, reduced mRNA expression of COL1A1 due to UVB treatment was significantly increased to a level comparable to that of vitamin C, which was used as the positive control (Figure 3C). Consistent with these results, Western blot results also showed an increase in MMP-1 expression and a decrease in COL1A1 levels after UVB irradiation; however, treatment of HDFs with the EA fraction reversed this expression (Figure 3D–F). These results indicate that the EA fraction inhibits the UVB-induced expression and secretion of MMP-1, resulting in the upregulation of COL1A1 levels in HDF cells.

### 2.4. Effect of EA Fraction on Activation of MAPK and NF-κB/AP-1 Signaling Pathways in UVB-Irradiated HDF Cells

**Figure 4:**
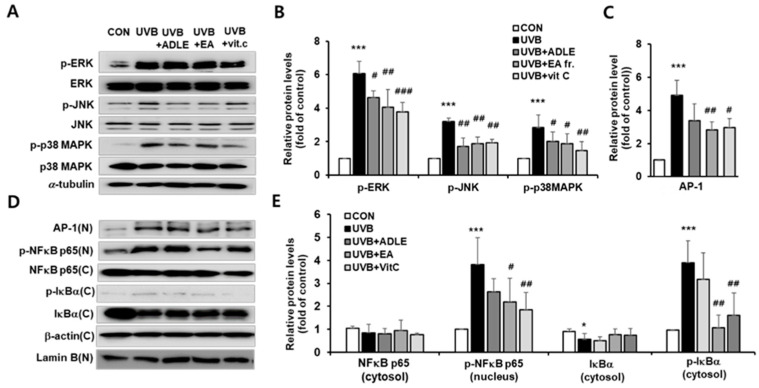
Effects of the EA fraction on the activation of MAPK and NF-*κ*B/AP-1 signaling pathways in UVB-irradiated HDF cells. Cells were treated for 24 h with 100 µg/mL of ADLE, 100 µg/mL of EA, and 10 µM of vitamin C after 100 mJ/cm^2^ UVB irradiation. The protein levels of MAPK pathway signals were analyzed using Western blotting (**A**), the band intensities were measured using C.S. analyzer 4 version 2.4.5 software, and relative quantities were calculated over α-tubulin (**B**). The protein levels of NF-κB related signaling and AP-1 (**C**) were analyzed using Western blotting (**D**), the band intensity was measured using C.S. Analyzer 4 version 2.4.5 software, and the relative quantity was calculated over β-actin and lamin B (**E**). Data are presented as mean ± SD (n = 3–5). * *p* < 0.05 and *** *p* < 0.001 versus CON. ^#^ *p* < 0.05, ^##^ *p* < 0.01, and ^###^ *p* < 0.001 versus UVB treatment alone. UVB cells were irradiated with 100 mJ/cm^2^ of UVB alone; UVB + ADLE cells were treated with 100 μg/mL of ADLE after UVB irradiation; UVB + EA cells were treated with 100 μg/mL of the EA fraction of ADLE after UVB irradiation; UVB + vitamin C cells were treated with 10 μM of ascorbic acid as a positive control.

To clarify the mechanism of EA-mediated inhibition of MMP-1 expression, the activation of MAPK was assessed via Western blotting. As shown in Figure 4A,B, phosphorylation of several MAPK family proteins, including ERK (*p* < 0.001), p38MAPK (*p* < 0.001), and JNK (*p* < 0.001), was significantly increased after UVB irradiation, which is consistent with a previous report [22]. In contrast, EA treatment resulted in the suppression of phosphorylation (Figure 4A,B). Following activation, the MAPK signaling pathways sequentially stimulate oxidative stress-induced signaling pathways such as NF-κB, transcription factor activator protein-1 (AP-1), and nuclear factor erythroid-2-related factor 2 (Nrf2) [23]. NF-kB/AP-1 is involved in the expression of MMP. We found that UVB irradiation significantly increased the translocation of AP-1 (*p* < 0.001) and p-NF-κB p65 (*p* < 0.001) from the cytoplasm into the nucleus, but treatment with the EA fraction markedly reduced the translocation of these proteins (*p* < 0.01) (Figure 4C). Next, we measured the changes in p-IκBα levels in the cytoplasmic fraction, which activates NF-κB through the dissociation of IκBα from the NF-κB complex. The cytoplasmic fraction of UVB-stimulated HDFs exhibited higher levels of p-IκBα than found in unstimulated cells. However, these levels were significantly suppressed following treatment with the EA fraction and were similar to those observed with vitamin C treatment (Figure 4D,E).

### 2.5. Inhibitory Effects of the EA Fraction on UVB-Induced Collagen Degradation in a 3D Reconstructed Human Skin Model

**Figure 5:**
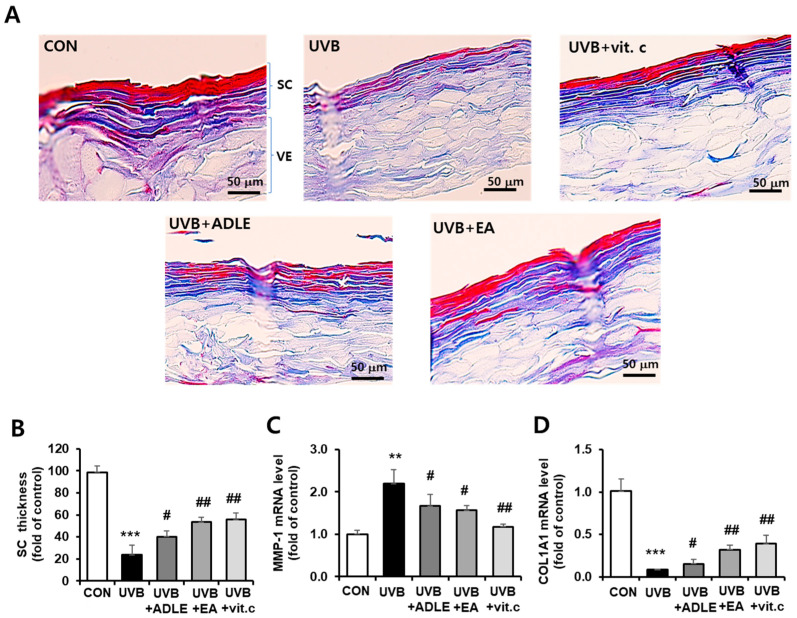
Inhibitory effects of the EA fraction on UVB-induced collagen degradation in a 3D reconstructed human skin model. Collagen deposition in the artificial epidermis model was observed at 400× magnification after Masson’s trichrome staining (**A**). This staining method produces red keratin, blue collagen, light-red or pink cytoplasm, and dark brown-to-black cell nuclei. Thickness of the stratum corneum (SC) was measured in the red- and blue-stained areas at the top of the membrane in three randomly selected fields in each epidermis (**B**). MMP-1 (**C**) and COL1A1 (**D**) mRNA levels were determined using *q*RT-PCR. The mRNA levels were normalized with those of cyclophilin. Data are presented as the mean ± SD (n = 3). ** *p* < 0.01 and *** *p* < 0.001 versus CON. ^#^ *p* < 0.05 and ^##^ *p* < 0.01 versus UVB alone. SC, stratum corneum layer; VE, viable epidermis layer; CON, nonirradiated control. UVB cells were irradiated with 100 mJ/cm^2^ of UVB alone; UVB + ADLE cells were treated with 100 μg/mL of ADLE after UVB irradiation; UVB + EA cells were treated with 100 μg/mL of the EA fraction of ADLE after UVB irradiation; UVB + vit. c cells were treated with 10 μM of ascorbic acid as a positive control.

To assess the histological changes in UVB-induced skin aging with or without EA-fraction treatment, EpiSkin^TM^ tissues from each group were stained with specific antibodies. First, Masson’s trichrome staining was used to visualize keratin (red) and collagen (blue) within the layers of the epidermis. Keratin has a unique form found only in the granular layer, which prevents moisture from evaporating from inside the skin and acts as a barrier against the penetration of foreign substances, particularly water. As shown in Figure 5A, red-stained keratin and blue-stained collagen were markedly decreased by UVB irradiation in the 3D skin model; however, EA treatment significantly reversed the loss of collagen and keratin within the stratum corneum, as evidenced by the upper cross-sectional structural thickness in the skin epidermis model (Figure 5B). Because the expression levels of COL1A1 and MMP-1 were regulated by EA-fraction treatment in HDF cells, we confirmed this effect in EPI skin tissues. COL1A1 mRNA was decreased by >90% following UVB irradiation (*p* < 0.001), whereas EA treatment restored COL1A1 mRNA levels 3.8-fold (*p* < 0.01), compared with the UVB-irradiated group alone. Additionally, MMP-1 mRNA, which increased 2.0-fold (*p* < 0.001) following UVB irradiation, was significantly suppressed by EA treatment (Figure 5C,D). These results indicate that the EA fraction inhibits UVB-induced skin barrier dysfunction and collagen reduction.

## 3. Discussion

Cutaneous aging is a complex biological process consisting of intrinsic and extrinsic aging. Intrinsic aging is a consequence of physiological and genetic changes that occur over time, whereas extrinsic aging is caused by cumulative exposure to external stimuli, such as UV radiation, environmental toxins, and infectious agents that cause DNA damage and, ultimately, skin damage [24]. The increase in UVB radiation is a major threat to skin health, with increased long-term consequences such as photoaging, photoimmunosuppression, and photocarcinogenesis [25].

UVB exposure increases ROS production, and excessive intracellular ROS production reduces mitochondrial membrane potential (ΔΨm), an indicator of electron transport chain homeostasis, thereby reducing ATP production [26]. Following the loss of ΔΨm, apoptosis-inducing factors, such as cytochrome c, are released from the mitochondrial intermembrane space into the cytoplasm and activate the caspase signaling pathway, resulting in apoptosis [27]. We demonstrated that UVB significantly increases intracellular ROS production and interferes with mitochondrial ATP synthesis, thereby inducing cell damage. However, treatment with ADLE and its EA fraction significantly reduced these effects, which was similar to the reduction observed with vitamin C. Although the antioxidant effects of the EA fraction are unclear, we hypothesize that they participate in regulating the oxidative system in photoaged fibroblasts through the induction of the cytoprotective enzymes HO-1 and NQO-1, which are downstream of Nrf-2.

Beta-galactosidase is a lysosomal hydrolase that cleaves terminal β-D-galactose residues [28,29]. As cellular aging progresses, or following UVB exposure, its activity gradually increases; therefore, it is widely used to assess the degree of cellular aging [30]. In this study, we confirmed the potential of EA as an inhibitor of β-galactosidase activity, which is activated during the photoaging process.

Cell-cycle arrest during cellular aging is primarily associated with the activation of one or both of the p53/p21^WAF1/CIP1^ and p16^INK4A^/pRB tumor-suppressor pathways. The activity of these pathways increases during the aging process [31,32]. Moreover, UVB primarily acts on the outermost epidermal layer of the skin. It causes an inflammatory response through the secretion of cytokines and chemokines, which contribute to inflammatory skin aging [33]. The expression of the mRNA or protein of cell cycle-related genes was increased by UVB and decreased by EA treatment. These results indicate that EA fractions not only delay the progression of cellular aging but also have an anti-inflammatory effect.

Collagen is the most widespread and abundant protein in the dermis and is one of the main ECM components responsible for skin elasticity and support [34]. MMPs are responsible for the degradation of ECM components in the dermis [35]. MMP-1 is increased by oxidative stress, such as UV, and partially cleaves collagen, whereas MMP-3 and MMP-9 break down the cleaved collagen fragments into smaller fragments. This destruction of connective tissue causes wrinkling and dryness of the skin, ultimately leading to photoaging [36]. Therefore, preventing collagen degradation by reducing MMP-1 activity may be an alternative to preventing photoaging. The EA fraction inhibited UVB-mediated expression of MMP-1 and increased COL1A1, which are related to the decomposition/synthesis of collagen in fibroblasts.

UV rays activate epidermal growth factor receptors and cytokine receptors on the surface of keratinocytes and fibroblasts, which in turn activate the ERK, JNK, and MAPK pathways. AP-1 acts as a downstream effector in these signaling pathways, leading to collagen degradation by upregulating MMPs. Therefore, inhibiting AP-1 activation is considered a rational approach to prevent skin aging [37,38,39]. Moreover, the abnormal accumulation of intracellular ROS is affected not only by the MAPK/AP-1 signaling pathway but also by nuclear factor-κB (NF-κB) signaling [40]. We confirmed that UVB irradiation activates ERK, JNK, and p38MAPK pathways, which activate AP-1 and NF-κB in the nucleus, increase MMP expression, and subsequently increase COL1A1 degradation. In the present study, activated MAPK/AP-1 and NF-κB signaling induced by UVB was attenuated by the EA fraction, suggesting that EA prevents collagen degradation by modulating UVB-induced MMP and proinflammatory cytokine expression through MAPK signaling mediated by the NF-κB p65 and AP-1 pathways in fibroblasts. Adler et al. showed that blocking the genetic induction of NF-κB in the epidermis of old mice restored tissue properties and the overall gene expression programs of young mice [41].

Reconstructed 3D human tissue models are widely used to examine the effects of cosmetic ingredients and their safety under in vivo-like conditions [42]. In the present study, the anti-aging effects of the EA fraction were examined using a 3D reconstructed human skin model. Similar to the HDFs, UVB irradiation decreased collagen levels and increased MMP levels, which were suppressed by the EA fraction.

Insects are recognized as a potential food resource. Although the uses of insects are derived primarily from unproven folk medicine, this resource has recently emerged as an industrial resource, resulting in studies of insect-derived physiological ingredients. The physiological substances identified in insects can be manufactured by the metabolism of phytophagous insects and synthesized during the ecdysis and sclerotization of insects [43,44]; however, the antioxidant effects of insect-derived compounds that target skin aging are unknown. Polyphenol compounds exhibit anti-inflammatory and anti-aging effects on the skin because of their excellent anti-inflammatory, antibacterial, and UV-blocking properties [45]. In a previous study, the total polyphenol content of the EA fraction extracted from ADLE was 10.3 mg GAE/g extract, which was 1.2-fold higher, compared with that of the raw material (ADLE). Therefore, EA contains anti-oxidant components derived from other solvent fractions, suggesting that it has an excellent antiphotoaging effect.

## 4. Materials and Methods

### 4.1. Chemicals and Reagents

Dulbecco’s modified eagle’s medium (DMEM), Trypsin-EDTA 1X solution, Dulbecco’s phosphate-buffered saline (DPBS), and penicillin–streptomycin were purchased from Welgene (Daegu, Republic of Korea). Fetal bovine serum (FBS) was purchased from Gibco BRL (Grand Island, NY, USA). Dimethyl sulfoxide (DMSO), Trizol reagent, and ascorbic acid were obtained from Sigma-Aldrich (St. Louis, MO, USA). Thiazolyl blue tetrazolium bromide (MTT) was obtained from Duchefa Biochemie (Haarlem, Netherlands). An oxidatively sensitive 2′,7′-dichlorodihydrofluorescein diacetate (DCFH-DA) fluorescent probe was obtained from Molecular Probes (Life Technologies, Grand Island, NY, USA).

### 4.2. Preparation of the EA Fraction of the ADLE

ADL were supplied by Yechun Bugs Land (Yecheon-gun, Gyungsangbuk-do, Republic of Korea). EA fractions of the aqueous ethanolic ADLE were prepared as previously described [19]. ADLE and EA fractions were prepared in sterile DMSO at a concentration of 100 mg/mL and diluted in culture medium to a final concentration of 100 μg/mL. The final DMSO concentration did not exceed 0.1% (*v*/*v*). Vitamin C, used as a positive control, was dissolved in distilled water.

### 4.3. Cell Culture, UVB Irradiation, and Cell Viability

Primary Dermal Fibroblast Normal; Human, Neonatal (HDFn, ATCC PCS-201-010), a cell line isolated from neonatal foreskin, is commonly used in research for studying responses to pathogens, skin aging, wound healing, gene delivery, and skin diseases, including scleroderma. Cells were proliferated and maintained in DMEM containing 10% FBS and 1% penicillin–streptomycin in a humidified incubator at 37 °C with 5% CO_2_ [46]. The culture medium was replaced every 3 days and passaged when the cells reached 80% confluence; cells with less than 25 passages were used for the experiment. UVB cells were irradiated at 100 mJ/cm^2^ using a UVB source (Bio-Link Crosslinker, Vilber Lourmat, Cedex, France). The cells were then treated with the indicated concentrations of the experimental samples for 24 h, and cell viability was measured using an MTT colorimetric assay.

### 4.4. Intracellular ROS Measurement

Intracellular ROS levels were detected using a DCFH-DA fluorescent probe, following the methods described previously [47]. Briefly, cells were grown to 80% confluence in a 96-well, black, clear-bottomed plate (Corning Inc., Corning, NY, USA), exposed to UVB irradiation, and incubated with ADLE, EA, and vitamin C for 24 h. The cells were washed twice with DPBS and reacted with DCFH-DA (10 µM) in the dark for 30 min. Thereafter, the cells were washed thrice with DPBS and 100 μL of DPBS was added to each well. The fluorescence values of dichlorofluorescein in the cells were detected using a fluorescence spectrophotometer at excitation and emission wavelengths of 488 and 535 nm, respectively.

### 4.5. Senescence-Associated β-Galactosidase Assay

HDF cells were incubated overnight in a 24-well plate, with 5.0 × 10^4^ cells per well. Confluent cells were irradiated with UVB and then treated with ADLE, EA, and vitamin C for 24 h. Senescence-associated β-galactosidase (SA-β-gal) levels were measured using a beta-galactosidase assay kit (Thermo Fisher Scientific, Waltham, MA, USA), following the manufacturer’s protocol.

### 4.6. ATP Content

Cellular ATP levels were measured based on luminescence using the Perkin–Elmer ATPLite system, following the manufacturer’s instructions. This was based on light production due to the ATP reaction with the added luciferase and D-luciferin. Briefly, cells were cleaned twice with DPBS after reaction termination. The mammalian cell lysis solution was then applied to release adenine nucleotides from cells and deactivate endogenous ATP-degrading enzymes. Then, luciferase and D-luciferin were added to react with the generated ATP.

### 4.7. Mitochondrial Membrane Potential Assay

A typical feature of apoptosis is the loss of mitochondrial membrane potential (ΔΨm). Intracellular mitochondrial membrane potential levels were measured based on fluorescence (ex. 550/em. 600 for red fluorescence and ex. 485/em. 535 for green fluorescence) using a JC-1 Mitochondrial Membrane Potential Detection Kit (Biotium, Hayward, CA, USA) following the manufacturer’s instructions. The results were expressed as the ratio of red fluorescence to green fluorescence. The ratio of red/green fluorescence was decreased in dead and apoptotic cells, compared with the ratio in healthy cells.

### 4.8. ELISA

HDF cells were seeded in a six-well plate at a density of 5.0 × 10^5^ cells/well and cultured for 24 h. The culture media and cells were collected after treatment of experimental samples for 24 h following UVB irradiation. The cultured media were centrifuged at 10,000× *g* for 3 min to remove debris, and the supernatants were collected. Quantitative measurement of MMP-1 secretion in the supernatant was performed using a Human MMP-1 (Sandwich ELISA) ELISA kit (catalog number ab215083, Abcam, Cambridge, MA, USA), following the manufacturer’s instructions. The collected cells were analyzed using the Cell Death Detection ELISA Plus kit (Roche Molecular Biochemicals, Mannheim, Germany) for the relative quantification of intracellular DNA damage (apoptotic). The protein content of the cells was determined using a bicinchoninic acid (BCA) protein analysis kit (Thermos Scientific, Rockford, IL, USA), with bovine serum albumin as the reference.

### 4.9. Nuclear Extract Preparation

Cells were treated with 100 mJ/cm^2^ UVB irradiation and then reacted with ADLE, EA, and vitamin C for 24 h. Cells were washed twice with DPBS, collected using a cell scraper, and pelleted at 12,000× *g* for 3 min at 4 °C. Cytosol and nucleic acids were separated from the collected cells according to the manual of NE-PER^®^ Nuclear and Cytoplasmic Extraction Reagents (Pierce Biotechnology, Rockford, IL, USA).

### 4.10. Treatment of a Human Reconstituted Skin Model and Measurement of Collagen Expression Using Histological Analysis and Gene and Protein Expression

Labcyte EPI-MODEL24 is a human reconstructed skin model purchased from Japan Tissue Engineering Co., Ltd. (J-TEC, Nagoya, Japan). Its structure is similar to that of the epidermis and comprises basal, spinal, granulous, and cornified layers. After receipt, the Labcyte EPI Model24 skin tissue was stabilized in a 5% CO_2_ incubator for 24 h. The upper surface of the well was then pretreated with an experimental sample diluted for 2 h in the provided assay medium, followed by three rinses with DPBS. Subsequently, the epidermal tissues dipped in 0.1-mL DPBS were irradiated using a UVB source (100 mJ/cm^2^) and then incubated in an assay medium diluted with experimental samples for 24 h. The epidermal tissues isolated from the specific insert plates were then collected for *q*RT-PCR and immunohistochemistry.

### 4.11. Masson’s Trichrome Stain

The following summarizes the paraffin embedding process: the culture inserts containing the EPI-MODEL were transferred to a tube containing 4% paraformaldehyde (PFA) and fixed for 24 h at 4 °C. The bottom layer was separated from the culture insert using a scalpel, and the upper layer tissue was embedded in a 4% agar solution dissolved in DPBS. This was performed using the paraffin embedding protocol for Masson’s trichrome staining [48].

### 4.12. Quantitative Real-Time Polymerase Chain Reaction

Total RNA sequentially extracted using Trizol reagent was reverse-transcribed using a PrimeScript cDNA Synthesis Kit (Takara, Dalian, China). The *q*RT-PCR was performed using SYBR Green Master Mix (Applied Biosystems, Foster City, CA, USA). The expression of each gene relative to cyclophilin, a reference gene, was calculated using the 2-ΔΔCt method. Table 1 shows the primer sequences.

### 4.13. Western Blotting

Western blotting was performed as previously described [19]. Proteins extracted from cells or skin tissue samples were transferred to nitrocellulose-based membranes after performing SDS-PAGE. The membranes were immunoblotted with the indicated primary antibodies overnight at 4 °C. After washing the membranes with TBST, they were reacted with secondary antibodies (Gendepot, Katy, TX, USA) at room temperature for 2 h. Chemiluminescence was performed using an ELC kit (Millipore, Burlington, MA, USA) and an ATTO WSE-6200 LuminoGraph II (ATTO Corporation, Tokyo, Japan). The images were evaluated using Image Software (C.S. Analyzer 4, ATTO Corporation, Tokyo, Japan) for quantitative analysis. The primary antibodies used for Western blotting were as follows: anti-AP-1 (1:1000; Cell Signaling Technology, Danvers, MA, USA), anti-MMP-1 (1:1000; Cell Signaling), anti-COL1A1 (1:1000; Cell Signaling), anti-p-ERK (1:1000; Cell Signaling), anti-ERK (1:1000; Cell Signaling), anti-p-JNK (1:1000; Cell Signaling), anti-JNK (1:1000; Cell Signaling), anti-p-p38MAPK (1:1000; Cell Signaling), anti-p38MAPK (1:1000; Cell Signaling), anti-p-NF-κB p65 (1:1000; Cell Signaling), anti-NF-κB p65 (1:1000; Cell Signaling), anti-p-IκB (1:1000; Cell Signaling), anti-IκB (1:1000; Cell Signaling), anti-COX2 (1:1000; Cell Signaling), anti-NQO-1 (1:1000; Abcam), HO-1 (1:1000; Abcam), anti-α-tubulin (1:1000; Cell Signaling), anti-β-actin (1:2500; Cell Signaling), and anti-Lamin B1 (1:1000; Cell Signaling).

### 4.14. Statistical Analysis

Statistical analysis was performed using the SPSS 20.0 software (IBM SPSS ver. 20.0.0 for Windows; IBM Co., Armonk, NY, USA). Results are expressed as the mean ± standard deviation. The significance of differences among groups was analyzed using the Fisher’s Least Significant Difference (LSD) comparison test. Statistical significance was set at *p* < 0.05.

## 5. Conclusions

**Figure 6:**
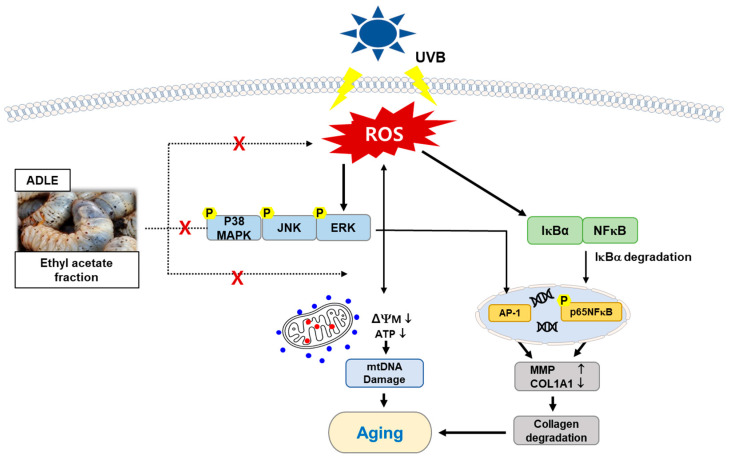
Scheme of the anti-aging mechanism of the EA fraction of ADLE on UVB-induced HDF cells. Red cross indicates that EA fraction of ADLE inhibits the pathways induced by UVB.

The EA fraction attenuated aging-related biochemical markers and prevented cell damage by downregulating the expression of cell-cycle arrest and inflammatory factors through the suppression of UVB-induced oxidative stress. Moreover, this effect was achieved through the control of MMPs through the MAPK/AP-1 and NF-κB signaling pathways (Figure 6). These results suggested that ADLE, especially its EA fraction, can more effectively prevent or ameliorate skin photoaging caused by UVB. These results provide insight for future anti-aging studies using insect materials.

## Figures and Tables

**Table 1:** Primer sequences.

Gene	Primer	Sequence (5′→3′)
*MMP-1*	forward reverse	ATTCTACTGATATCGGGGCTTTGA ATGTCCTTGGGGTATCCGTGTAG
*MMP-3*	forward reverse	TTCGGGATGCCAGGAAAGGTTC AGTTCCTTGGATTGGAGGTGACG
*MMP-9*	forward reverse	CTGCCAGGACCGCTTCTACT TGGTCCCAGTGGGGATTTAC
*COL1A1*	forward reverse	CTCGAGGTGGACACCACCCT CAGCTGGATGGCCACATCGG
*TNFα*	forward reverse	TGCTCCTCACCCACACCAT GGAGGTTGACCTTGGTCTGGTA
*IL-1β*	forward reverse	ACGATGCACCTGTACGATCACT CACCAAGCTTTTTTGCTGTGAGT
*IFNγ*	forward reverse	ACTCATCCAAGTGATGGCTGAA TCCTTTTTCGCTTCCCTGTTT
*p16*	forward reverse	GTGGACCTGGCTGAGGAG CTTTCAATCGGGGATGTCTG
*p21*	forward reverse	CCGAAGTCAGTTCCTTGTGG CATGGGTTCTGACGGACAT
*p53*	forward reverse	AGGCCTTGGAACTCAAGGAT CCCTTTTTGGACTTCAGGTG
*GAPDH*	forward reverse	AGGGCTGCTTTTAACTCTGGT CCCCACTTGATTTTGGAGGGA

## Data Availability

The data used to support the findings of this study are available from the corresponding author upon request.
